# Construct validity of EQ-5D-5L among patients with inflammatory bowel disease—a study based on real-world data from the Swedish Inflammatory Bowel Disease Registry

**DOI:** 10.1186/s41687-024-00709-9

**Published:** 2024-03-27

**Authors:** Jack Latteur, Olivia Ernstsson, Evalill Nilsson, Susanna Jäghult, Emelie Heintz

**Affiliations:** 1 Health Economic and Policy Research Group, Medical Management Centre, Department of Learning, Informatics, Management and Ethics (LIME), Karolinska Institutet, Stockholm, Sweden; 2 Health Economics and Economic Evaluation Research Group, Medical Management Centre, Department of Learning, Informatics, Management and Ethics (LIME), Karolinska Institutet, Stockholm, Sweden; 3 eHealth Institute, Department of medicine and optometry, Linnaeus University, Kalmar-Växjö, Sweden; 4 Department of Clinical Sciences, Karolinska Institutet, Danderyd Hospital, Södersjukhuset, Stockholm, Sweden; 5 Stockholm Center for Health Economics, Center for Health Economics, Informatics and Health Services Research (CHIS), Stockholm Healthcare Services, Stockholm, Sweden

**Keywords:** Inflammatory bowel disease, Ulcerative colitis, Crohn’s disease, EQ-5D-5L, PROMs, Construct validity, Known-groups validity, Convergent validity

## Abstract

**Objectives:**

The Swedish Inflammatory Bowel Disease Registry (SWIBREG) includes approximately 84% of all patients with inflammatory bowel disease (IBD) treated with immunomodulators, biologics or surgery in Sweden. Data on health-related quality of life (HRQoL) have been collected using EQ-5D-5L in the registry since 2012. Nevertheless, there are few studies assessing the validity of EQ-5D-5L in this patient population. Thus, the aim of this study was to assess the construct validity of EQ-5D-5L amongst patients with IBD (ulcerative colitis and Crohn’s disease).

**Methods:**

Individual-level data on EQ-5D-5L and other disease-specific measures were extracted from SWIBREG. Known-groups validity was assessed by analysing whether the EQ-5D-5L captured expected differences between patient groups with different activity levels of the disease. Convergent validity was assessed by analysing whether the reported problems in the dimensions of EQ-5D-5L, EQ VAS, and the EQ-5D-5L index value correlated, as hypothesized, with the four dimensions in the Short Health Scale, a symptom index question, and the Physician Global Assessment (PGA) score.

**Results:**

In total, 9769 patients with IBD were included in the study. Patients with active IBD reported more health problems in the EQ-5D-5L descriptive system than patients being in remission. The effect sizes for the differences in reported problems between patients with active and inactive disease were at least small (≥0.1) or medium (≥0.3) in all dimensions except self-care. Differences in the mean EQ-5D-5L index and EQ-VAS score between patients with active and inactive disease were statistically significant (*p* < 0.001) and larger than pre-defined cut-offs for minimally important differences (>0.08 for the index and >11.0 for EQ-VAS). The analysis of convergent validity showed that EQ-5D-5L results correlated as expected with the disease-specific measures in 16 of the 21 analyses. In total, 22 (79%) of the 28 hypotheses were supported.

**Conclusion:**

The findings support the construct validity of EQ-5D-5L amongst patients with IBD and contribute to the scarce literature on the validity of the five-level version of EQ-5D in this patient population. These findings have important implications for the choice of HRQoL measure in routine health care registries like SWIBREG as well as for future clinical or health economic studies considering using EQ-5D-5L as a measure of HRQoL.

## Introduction

Inflammatory bowel disease (IBD) is a chronic bowel disorder that can manifest as two main types, Crohn’s Disease (CD) and Ulcerative Colitis (UC), which are characterized by inflammation of the gastrointestinal tract [1, 2]. While UC affects the colon, CD may affect all parts of the gastrointestinal tract. The symptoms of both types are similar and include bloody diarrhoea, abdominal pain, rectal bleeding, weight loss, and fatigue [1–3]. The prevalence of IBD was reported at 6.8 million cases worldwide in 2017 and evidence shows that this estimate has been increasing across the globe [4]. Prevalence in Sweden was estimated at just over 60,000 people in 2010, representing around 0.65% of the total Swedish population [5].

IBD can significantly reduce the quality of life of patients, through both physical pain and social discomfort [6–10]. There are various disease-specific and generic measures to estimate this impact on quality of life. One example of such a measure is the EQ-5D, which is a generic patient-reported outcome measure (PROM) designed to be applicable to many different diseases and conditions [11, 12]. It was designed to provide a standardised measure of health-related quality of life (HRQoL), which could be used in clinical studies and economic evaluation. Currently, it is the most frequently recommended measure of HRQoL when conducting economic evaluations [13]. Nevertheless, its use in clinical outcome assessment has been limited [14]. Among IBD patients, EQ-5D has, to some extent, previously been used to evaluate and quantify the patients’ HRQoL [9, 10]. The instrument has also been implemented in several Swedish national quality registers across many different patient populations and conditions, for example to assess the self-reported health status and/or for follow-up of interventions [15]. In the Swedish Inflammatory Bowel Disease Registry (SWIBREG), EQ-5D-5L data has been collected since 2012 [15, 16].

The EQ-5D questionnaire consists of two parts that examine how the respondents perceive their health: a descriptive system with five items and a rating of overall health [11, 12]. The five items each represent one dimension of health (i.e., mobility, self-care, usual activities, pain/discomfort, anxiety/depression). For adults, there are currently two versions of the questionnaire: the EQ-5D-3L, in which each dimension has three response levels, and the newer EQ-5D-5L which has five response levels for each dimension. The EQ-5D-5L version was introduced to combat concerns regarding the sensitivity of the original version that only had three response levels (EQ-5D-3L) [17].

To ensure that the results from an outcome measure are useful, the measure must be found to be valid [18]. Validity is defined as the extent to which an instrument accurately measures what it intends to measure [18]. Assessing the validity of measures of HRQoL is important to ensure that the instruments used in health care registers or clinical studies can accurately describe the patients’ HRQoL and can detect meaningful differences in HRQoL between patients [19]. When measuring constructs that are not directly observable, such as HRQoL, it is recommended to focus on the construct validity of the instrument [18, 20–22]. Construct validity is assessed by analysing the degree to which an instrument appears to measure the construct that it was designed to measure [18]. It can be assessed by analysing known-groups validity and convergent validity. Known-groups validity is examined by testing whether a measure can detect expected differences between patient groups and/or other subgroups [18]. Convergent validity is investigated by testing if the results from an instrument correlate with those of other instruments that are theoretically similar.

To the authors’ knowledge, the validity of EQ-5D amongst IBD patients has been assessed in three previous studies [23–25]. Two of the studies were conducted in Germany and investigated the construct validity of EQ-5D-3L by analysing the correlation between EQ-5D-3L and disease activity indices, and differences in EQ-5D scores between patients with inactive and active disease [24, 25]. The results supported the validity of the EQ-5D-3L index and EQ VAS among patients with IBD. The third study compared the construct validity of EQ-5D-3L and EQ-5D-5L among patients with CD in Hungary. In that study, the findings regarding the relationship between the results from EQ-5D-5L and patient-reported scales supported construct validity of the two versions of the instrument [23]. Nevertheless, there was a weaker relationship between the two versions of EQ-5D and physician-reported measures of disease severity. All three studies were based on relatively small sample sizes (152–502 patients) and no study has to our knowledge investigated the validity of EQ-5D-5L among patients with UC. Thus, this study aimed to investigate the construct validity of EQ-5D-5L amongst patients with IBD (UC and CD) in a national sample from clinical settings in Sweden.

## Methods

### Study design and data source

The study was a cross-sectional register-based study assessing the construct validity of the EQ-5D-5L amongst patients with IBD. Individual-level data on the EQ-5D-5L and other relevant variables were extracted from SWIBREG. SWIBREG was created in 2005 and contains data for patients of all ages with CD, UC and unclassified IBD (IBD-U). In 2021, more than 84% (>57,000 individuals) of all patients with IBD treated with immunomodulators, biologics or surgery in Sweden were included in the registry [26]. SWIBREG contains sociodemographic data as well as disease-specific information such as diagnosis, year and age of diagnosis, disease activity, examinations, and treatment. Furthermore, it contains data from various PROMs, such as the EQ-5D-5L and the Short Health Scale (SHS). The current study was approved by the regional ethical board in Stockholm (DNR 2018/1137-31/2).

### Study population

**Fig. 1:**
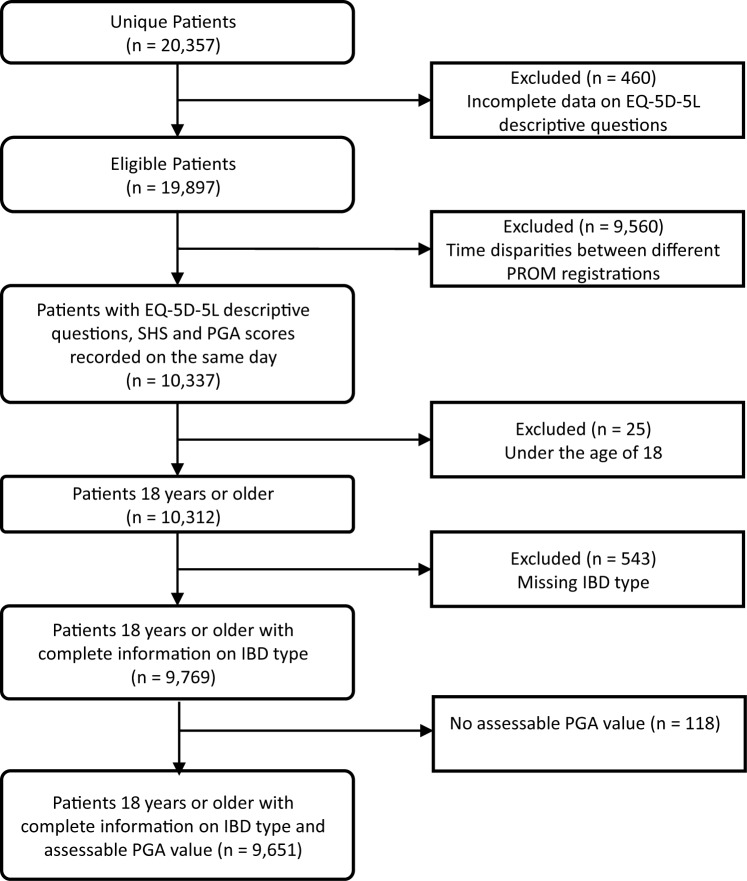
Flow chart describing the sample included in the study

The study sample included all patients from SWIBREG 18 years or older, with complete data, and registrations at the same point in time, for the EQ-5D-5L descriptive system, SHS, and Physician Global Assessment (PGA) (Fig. 1). The sample covered patients with EQ-5D-5L registrations from 9th February 2012 to 16th March 2021. Only one EQ-5D-5L registration per patient was used in the analysis. In the case that patients had several complete registrations of the EQ-5D-5L, SHS, and the PGA score, the most recent registration was chosen.

### Measures

The variables that were included in this study were background characteristics (age at diagnosis, age at EQ-5D registration, sex), Physician Global Assessment (PGA), PROMs (EQ-5D-5L and SHS), and clinical information pertaining to the patients’ disease (symptom index scores).

#### EQ-5D-5L

The first part of the EQ-5D is a questionnaire that contains five items, each representing a dimension of health (mobility, self-care, usual activities, pain/discomfort, anxiety/depression) [11, 12]. The five response levels for each dimension are: no problem (1), slight problems (2), moderate problems (3), severe problems (4) and unable/extreme problems (5) [17]. The responses generate a five-digit code describing their health state (the descriptive health profile). For example, the numerical health profile for no problems in any dimension is 11111. From the health profile, an index value can be calculated. The index value is a single number that represents the value of being in a particular health state. The index values are calculated using predefined value sets with algorithms to calculate index values for all possible EQ-5D five-number outputs. For the index values in this study, a crosswalk value set based on a value set for EQ-5D-3L from the UK was used in our base-case analysis [27]. The index values in this value set range between −0.59 and 1. To investigate the influence of the choice of value set, two Swedish value sets were used in a sensitivity analysis [28, 29]. The most recent of the two (published after the initiation of this study) is based on a valuation protocol (EQ-VT) developed by the EuroQol Research Foundation [28, 30]. The other is based on responses from a survey in which the respondents completed the EQ-5D-5L and valued their own health with an open-ended time trade-off (TTO) question [29]. The second part of EQ-5D-5L consists of a visual analogue scale (EQ VAS) in which patients rate their health today on a scale ranging from 0 to 100, with 0 representing the worst imaginable health state and 100 representing the best imaginable health state.

#### Short Health Scale (SHS)

The SHS was developed as a measure of HRQoL in patients with CD and UC [31, 32]. It was developed in Sweden and comprises four questions intended to assess symptom severity, functional status (the impact of the disease on daily life), the worry patients experience, and the patient’s self-perception of their wellbeing. The Swedish and English versions of the measure have been validated for use in patients with IBD [31–33]. For each of the four questions, SWIBREG uses a scale with six response levels [16].

#### Physician Global Assessment

The Physician Global Assessment (PGA) is an overall measure of disease activity based on the physicians’ assessment of recorded symptoms, findings from endoscopic examinations, and other observations, such as physical findings and the patients’ performance status [34, 35]. It is included as one of the four parts of the Mayo score, which is a commonly used disease activity index in clinical trials [36, 37]. The results from the PGA are presented as one of four categories: inactive disease (0), mild disease activity (1), moderate disease activity (2) or severe disease activity (3). The PGA is used by SWIBREG as a quality indicator with the goal that more than 50% of the patients should have a registered PGA value and that 90% of the patients should be in no or mild activity [16, 38]. To guide the assessment of disease activity, the physicians reporting to SWIBREG assess the following aspects: abdominal pain, stool form, fatigue, activity, presence of fistula or other perianal disease, weight loss, abdominal tenderness, morbid appearance, laboratory tests [39].

#### Symptom index scores

SWIBREG also contains a range of patient-reported symptom index scores to assess and document the patients’ symptoms. These index scores measure factors such as physical symptoms, feelings of pain, and wellbeing. In this particular study, the symptom index questions “Have you had abdominal pain?” and “How is your general well-being?” were considered relevant for the analyses. However, the question about well-being was excluded as it was very similar to the question about well-being in the SHS.

### Assessment of construct validity

The construct validity of EQ-5D-5L was assessed by analysing known-groups validity and convergent validity. In line with COSMIN guidelines on the study design for the evaluation of measurement properties of PROMs, the analyses of known-groups validity and convergent validity are based on tests of predetermined hypotheses regarding the relationships between the EQ-5D-5L variables and other measures [20]. The hypotheses were formulated based on previous literature and discussions among the authors regarding the similarity of the constructs measured by the instruments. In all relevant analyses, an alpha value of 0.05 was selected. Following the Cosmin guidelines, at least 75% of the hypotheses were required to be supported to conclude that the findings of the study show support for construct validity of EQ-5D-5L [40, 41].

#### Known-groups validity

Known-groups validity was assessed by testing whether the EQ-5D-5L captured expected differences between patient groups. The groups were based on the activity of the disease as defined by the PGA score. It was expected that patients with more active disease would report more problems and score their overall HRQoL as worse than those with less active disease. Three hypotheses were formulated for this analysis:

- Patients with higher PGA scores (more active disease) have worse HRQoL than patients with lower PGA scores (less active disease) and will therefore score lower on the EQ-5D-5L index (Hypothesis 1).
- Patients with higher PGA scores (more active disease) have worse HRQoL than patients with lower PGA scores (less active disease) and will therefore score lower on the EQ VAS (Hypothesis 2).
- Patients graded as having active IBD (PGA = 1, 2, 3) would report more problems on the five EQ-5D-5L dimensions than those graded as being in remission (PGA = 0) (Hypothesis 3–7, one per dimension).

Differences were assessed based on statistical significance and the size of the differences between groups. To guide the assessment of the size of mean differences in the EQ-5D index value and EQ VAS, minimally important differences of 0.08 and 11.0 from a previous study among patients with IBD were used as reference points for the EQ-5D-5L index and EQ VAS respectively [25].

To test hypothesis 1 and 2 mean EQ-5D-5L index and EQ VAS scores were compared between patients with active and inactive disease. An independent *t*-test was performed to test if the differences were statistically significant. To get a more detailed understanding of the ability of EQ-5D-5L to detect differences between the activity levels, further comparisons of the EQ-5D-5L index and EQ-VAS were conducted between patients in the four activity levels of the PGA. A one-way ANOVA test was run to compare the EQ-5D-5L index value and EQ-VAS of the four different PGA groups. However, when running the test of homogeneity of variances, significant values below 0.001 were registered, implying a violation of the assumption of homogeneity of variances. Therefore, rather than using the test statistic and significance value from the ANOVA output, a Brown-Forsythe test was run.

To test hypotheses 3–7, the distributions of responses across the five response levels for each dimension of the EQ-5D-5L were compared between patients with active disease and inactive disease. Due to the categorical characteristic of the data and the groups of patients with active and inactive disease being independent, a Mann-Whitney U test was used to test for statistical differences in the distribution of responses [42]. A rough estimate of the effect size (r) was calculated using the absolute Z scores from the Mann-Whitney U test and the number of total cases (n) in the following formula: $r=\frac{{z}}{{\sqrt{n}}}$ [43]. The test statistic r is the degree of difference between the groups that are being tested. The effect size was considered small if between 0.1 and 0.29, medium if between 0.3 and 0.49, and large if 0.5 and above [44].

#### Convergent validity

Convergent validity was assessed by testing expected relationships between EQ-5D-5L and other outcome measures [20]. Thus, hypotheses regarding the correlation between the results from the descriptive system of the EQ-5D-5L, EQ VAS, and the EQ-5D index, and the results from the four items in SHS, the symptom index about abdominal pain, and the PGA score were formulated and tested. The hypotheses for the analysis of convergent validity included predictions on the direction and strength of the correlations between EQ-5D-5L and other IBD-specific measures based on their theoretical similarities (the hypotheses are presented in Table 1). Constructs that were considered related were expected to have a correlation of 0.3 or more (interpreted as moderate) while constructs that were considered similar were expected to have a correlation of at least 0.5 (interpreted as strong) [40, 45]. Because the variables with which the EQ-5D was compared are categorical, the non-parametric Spearman rank correlation coefficient was used for all correlation tests in the analysis of convergent validity [42].

**Table 1:** Expectations on directions and strengths of the correlations between EQ-5D-5L and the other measures in the analysis of convergent validity

	Mobility	Self-care	Usual activities	Pain/discomfort	Anxiety/depression	EQ VAS	EQ-5D-5L index
Hypothesis 8–14	H8	H9	H10	H11	H12	H13	H14
PGA	++	++	++	++	++	−−−	−−−
Hypothesis 15–16						H15	H16
SHS 1 (Severity of symptoms)						−−−	−−−
Hypothesis 17–18			H17			H18	H19
SHS 2 (Interference in daily life)			+++			−−−	−−−
Hypothesis 19–21					H20	H21	H22
SHS 3 (Worry caused by IBD)					+++	−−−	−−−
Hypothesis 22–24					H23	H24	H25
SHS 4 (General feeling of well-being)					+++	−−−	−−−
Hypothesis 25–27				H26		H27	H28
Symptom Index 7 (Abdominal pain)				+++		−−−	−−−

The plus and minuses represent the direction and the strength. ++: at least a positive correlation of 0.3 (moderate), +++: at least a positive correlation of 0.5 (strong). Negative symbols follow the same interpretation of the strength of the correlation but represent negative correlations

*H* Hypothesis, *PGA* Physician Global Assessment, *SHS* Short Health Scale, *IBD* Inflammatory bowel disease, *EQ VAS* Visual analogue scale in the EQ-5D-5L questionnaire

## Results

### Background characteristics

Of the 9769 patients included in the study, 4159 (40.3%) had CD, 5143 (49.9%) had UC, and 467 (4.5%) were classified as IBD-U (IBD unclassified) (Table 2). Age and sex distributions were similar amongst the two main IBD diagnosis groups. For CD and UC, mean age was 28.8 and 32.4 respectively and males made up 51.6% and 53.5% of the sample respectively. Average EQ-5D-5L index values and EQ VAS scores for patients with CD were 0.77 and 73.33 respectively. For patients with UC, the corresponding numbers were 0.80 and 75.73. These estimates for patients with CD and UC can be compared to averages of the Swedish general population, which have been reported to be around 0.88 for the EQ-5D-3L index among individuals 30–39 year old and 77.4–81.5 for EQ VAS among 30–34 years old women and men [29, 46]. The distribution of PGA scores was consistent across the two main IBD diagnosis groups with the majority of patients being defined as having inactive disease and the proportion of patients in each group decreasing as the PGA score increased. Out of the total sample, 118 patients had a PGA value recorded as not assessable (Fig. 1).

**Table 2:** Background characteristics

	Total n = 9769	Crohn’s disease n = 4159	Ulcerative colitis n = 5143	IBD-U n = 467
Sex				
Male	52.0%	51.6%	53.5%	38.3%
Female	48.0%	48.4%	46.5%	61.7%
Age at diagnosis, mean (SD)	31.28 (15.75)	28.81 (14.91)	32.35 (15.55)	41.53 (19.45)
Age at EQ-5D-5L registration, mean (SD)	46.94 (17.28)	46.27 (17.21)	47.21 (17.09)	50.02 (19.44)
PGA				
Inactive disease	67.5%	66.0%	69.0%	63.2%
Mild activity	19.4%	20.7%	18.0%	22.7%
Moderate activity	10.1%	10.0%	10.2%	10.1%
Severe activity	1.8%	1.3%	2.1%	2.8%
Not assessable	1.2%	1.9%	0.6%	1.3%
SHS symptoms				
No symptoms	30.0%	24.9%	34.6%	23.8%
Mild symptoms	32.6%	34.3%	31.6%	27.2%
Moderate symptoms	20.3%	23.2%	17.8%	23.1%
Rather severe symptoms	9.9%	10.6%	8.9%	15.0%
Severe symptoms	5.2%	5.1%	5.2%	7.3%
Very severe symptoms	1.9%	1.9%	2.0%	3.6%
SHS Interference in daily life (functional status)				
Not at all	33.4%	28.5%	37.3%	34.0%
To a low degree	30.8%	30.7%	31.3%	25.7%
To a moderate degree	17.4%	19.9%	15.5%	16.3%
To a fairly high degree	9.1%	10.4%	7.9%	11.1%
To a high degree	5.3%	5.8%	4.8%	6.9%
To a very high degree	4.0%	4.7%	3.2%	6.0%
SHS Worry due to IBD				
Not at all	20.0%	18.0%	21.5%	19.9%
To a low degree	34.2%	34.6%	34.4%	27.8%
To a moderate degree	22.8%	23.2%	22.5%	23.6%
To a fairly high degree	11.8%	11.9%	11.4%	15.0%
To a high degree	6.9%	7.7%	6.1%	7.3%
To a very high degree	4.4%	4.7%	4.0%	6.4%
SHS General well-being				
Very good	17.0%	15.4%	18.1%	18.8%
Good	36.1%	35.1%	37.8%	27.2%
Fairly good	28.3%	29.1%	27.7%	28.5%
Bad	12.7%	14.1%	11.1%	17.3%
Very bad	4.5%	5.0%	4.0%	5.8%
Terrible	1.4%	1.3%	1.3%	2.4%
SI: Abdominal pain, n^a^	8132	3491	4298	343
None	53.7%	47.8%	59.1%	46.4%
Mild	28.9%	31.1%	26.9%	31.2%
Moderate	13.0%	15.4%	10.8%	15.5%
Severe	4.4%	5.6%	3.2%	7.0%
EQ-5D-5L index, mean (SD)	0.79 (0.21)	0.77 (0.22)	0.80 (0.20)	0.76 (0.21)
EQ-5D-5L VAS, mean (SD)	74.48 (19.48)	73.33 (20.00)	75.73 (18.85)	70.56 (20.71)

*IBD* Inflammatory Bowel Disease, *IBD-U* IBD Unclassified, *PGA* Physician Global Assessment, *SD* Standard deviation, *SHS* Short Health Scale, *SI* Symptom index

^a^The number of patients who had reported SI Abdominal pain was lower as there were some missing values

### EQ-5D-5L descriptive system

**Fig. 2:**
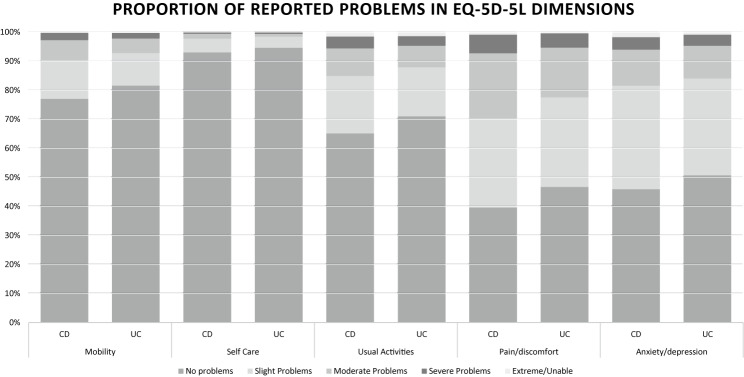
Responses in the five dimensions of the EQ-5D-5L descriptive system among patients with Crohn’s disease (CD) and patients with Ulcerative colitis (UC)

The most frequently reported problems by patients were problems with pain/discomfort and with anxiety/depression (Fig. 2). The best possible health state in the EQ-5D-5L descriptive system (11111) was reported by 3038 patients in the sample, representing 29.5% of the entire sample.

### Known-groups validity

**Fig. 3:**
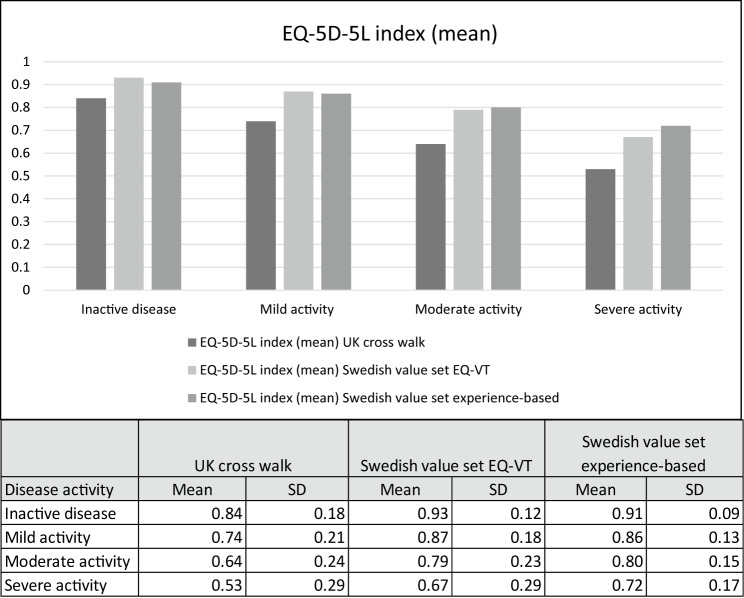
Mean EQ-5D-5L index values for patients with different levels of disease activity. Disease activity was defined according to the Physician Global Assessment (PGA) score. *SD* Standard deviation

**Fig. 4:**
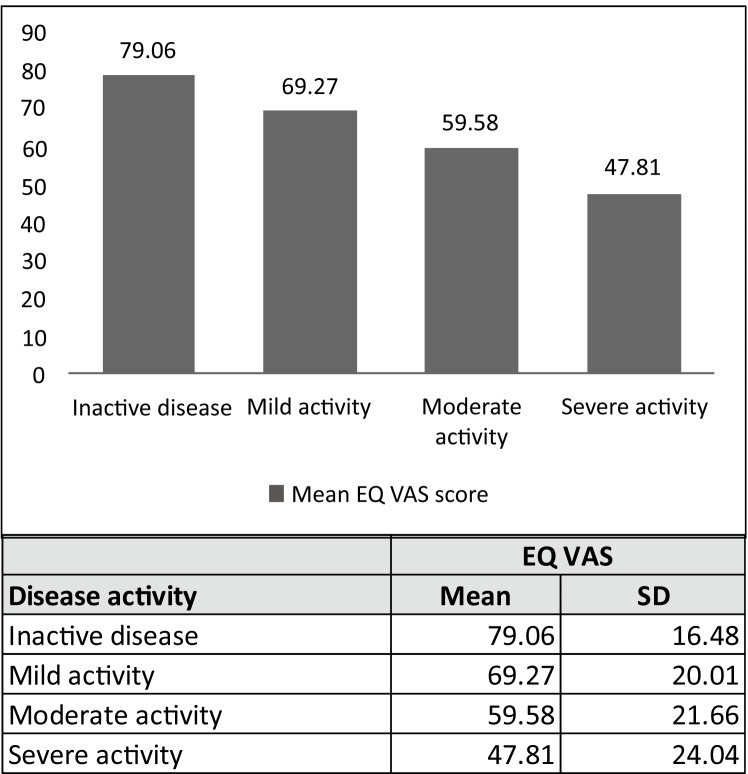
Mean EQ VAS scores for patients with different levels of disease activity. Disease activity was defined according to the Physician Global Assessment (PGA) score. *SD* Standard deviation

The mean difference in EQ-5D-5L index values (UK crosswalk) between patients in remission and in active disease was 0.16 for CD (0.83 vs. 0.67) and 0.14 for UC (0.85 vs. 0.71) (Table 3). The corresponding numbers for EQ VAS were 15.14 (78.50 vs. 63.36) and 13.13 (79.72 vs. 66.59) (Table 3). Thus, the differences in mean EQ-5D-5L index values and mean EQ VAS scores between the patient groups with active and inactive disease were equal to, or larger than, the predefined cut-offs for minimally important differences, which had been selected based on previous literature (0.08 for the index value and 11.0 for the EQ VAS) [25]. These results were stable when dividing the study population into groups of men and women. The differences were also found to be statistically significant (*p* < 0.001). Altogether, this indicates that the EQ VAS and the EQ-5D-5L index are able to capture differences between patients with active and inactive disease. Consequently, the first two hypotheses were supported. In the analysis of how the choice of value set influenced the results, the mean difference in the EQ-5D-5L index between patients with active and inactive disease was 0.10 (0.93 vs. 0.83, *p* < 0.001) when using the Swedish value set based on the EQ-VT protocol and 0.08 (0.91 vs. 0.83, *p* < 0.001) when using the Swedish experience-based value set. The mean EQ-5D-5L index values calculated based on the three value sets and the mean EQ VAS score for the four PGA groups are presented in Figs. 3 and 4. With the UK crosswalk value set, the differences in the mean EQ-5D-5L index value between the activity levels were larger than the cut-off for meaningful differences. With the other value sets, the differences were smaller but still statistically significant (*p* < 0.001).

**Table 3:** EQ-5D-5L index values, EQ VAS and the EQ-5D-5L descriptive system for patients with active and inactive IBD

		Crohn’s disease (n = 4080)			Ulcerative colitis (n = 5110)			Total (n = 9651)		
		Inactive n = 2747 (100%)	Active n = 1333 (100%)		Inactive n = 3550 (100%)	Active n = 1560 (100%)		Inactive n = 6592 (100%)	Active n = 3059 (100%)	
	Mean EQ-5D index	0.83	0.67	p<0.001	0.85	0.71	p<0.001	0.84	0.69	p<0.001
	Mean EQ VAS^a^	78.50	63.36	p<0.001	79.72	66.59	p<0.001	79.06	64.87	p<0.001
Mobility	No problems (%)	82.1	68.0	p<0.001 z = −10.5 r = 0.16	83.9	76.3	p<0.001 z = −6.6 r = 0.09	82.8	72.6	p<0.001 z = −12.0 r = 0.12
	Slight problems (%)	11.6	16.7		10.0	13.6		10.7	15.0	
	Moderate problems (%)	4.7	10.9		4.2	7.0		4.6	8.7	
	Severe problems (%)	1.6	4.1		1.6	2.8		1.6	3.4	
	Unable (%)	0.1	0.3		0.3	0.3		0.2	0.3	
Self-Care	No problems (%)	95.5	89.0	p<0.001 z = −7.9 r = 0.12	95.6	92.5	p<0.001 z = −4.5 r = 0.06	95.4	91.1	p<0.001 z = −8.3 r = 0.08
	Slight problems (%)	3.2	7.1		3.1	5.4		3.3	6.0	
	Moderate problems (%)	0.9	3.1		0.8	1.4		0.8	2.1	
	Severe problems (%)	0.3	0.7		0.5	0.6		0.4	0.6	
	Unable (%)	0.1	0.2		0.1	0.1		0.1	0.1	
Usual activities	No problems (%)	74.8	46.5	p<0.001 z = −18.8 r = 0.29	80.2	50.7	p<0.001 z = −22.3 r = 0.31	77.8	48.8	p<0.001 z = −29.9 r = 0.30
	Slight problems (%)	16.1	27.0		13.2	25.1		14.5	26.0	
	Moderate problems (%)	6.7	14.9		4.3	14.0		5.3	14.4	
	Severe problems (%)	1.7	8.9		1.6	7.0		1.7	7.8	
	Unable (%)	0.7	2.8		0.6	3.1		0.7	2.9	
Pain/discomfort	No (%)	49.9	19.7	p<0.001 z = −22.3 r = 0.35	57.2	23.5	<0.001 z = −23.6 r = 0.33	53.7	21.7	p<0.001 z = −32.9 r = 0.34
	Slight (%)	30.8	30.8		27.4	38.7		28.6	34.7	
	Moderate (%)	15.8	35.5		12.3	27.6		14.3	31.5	
	Severe (%)	3.1	12.3		2.9	9.4		3.1	10.7	
	Extreme (%)	0.3	1.8		0.2	0.8		0.3	1.4	
Anxiety/depression	Not (%)	54.5	29.4	p<0.001 z = −17.4 r = 0.27	59.8	30.3	<0.001 z = −21.5 r = 0.30	57.3	30.1	p<0.001 z = −28.0 r = 0.28
	Slightly (%)	33.6	40.4		30.2	40.6		31.6	40.4	
	Moderately (%)	9.1	18.4		7.4	19.9		8.3	19.3	
	Severely (%)	2.1	8.4		2.1	7.8		2.1	8.0	
	Extremely (%)	0.7	3.5		0.5	1.4		0.7	2.3	

EQ VAS n CD: 2 388 (inactive disease), 1 162 (active disease). EQ VAS n UC: 3 080 (inactive), 1 310 (active). EQ VAS n Total: 5 691 (inactive), 2 594 (active)

*r* effect size, *Z* Z-score Mann-Whitney U test, *p* *p*-value

^a^Due to missing values, the number of patients (n) with EQ VAS score were lower

In each of the five EQ-5D-5L health dimensions, patients with active disease reported statistically significantly more problems than patients with inactive disease (Table 3). For example, around 80 and 70% of patients with active CD reported problems with pain/discomfort and anxiety/depression respectively, while the corresponding percentages for patients with inactive CD were around 50 and 45%. Nevertheless, the effect sizes varied. For usual activities, pain/discomfort, and anxiety/depression, effect sizes were all medium or close to medium in the total population, as well as in the UC and CD subgroups (Table 3). However, the effect sizes for the differences in the distributions of responses in the mobility and self-care dimensions were smaller, at 0.12 and 0.08 in the total population, and even smaller for UC patients, at 0.09 and 0.06 respectively. Nevertheless, six (86%) of the seven hypotheses (hypothesis 1–3 and 5–7 of hypotheses 1–7) for known group validity were confirmed.

### Convergent validity

In most cases, the responses to the EQ-5D-5L descriptive system correlated with the other measures as hypothesized (Tables 1 and 4). The strength of the correlations between the EQ-5D-5L dimensions, EQ-VAS, and the EQ-5D-5L index, and the four SHS questions matched the expectations of a strong correlation (≥0.5) (hypotheses 15–25). As expected, the PGA had moderate correlation (≥ 0.3) with the reported problems with usual activities (r = 0.33), pain/discomfort (r = 0.35), and anxiety/depression (r = 0.30) (hypotheses 10–12). However, the correlations between PGA and some of the EQ-5D-5L variables (EQ VAS, the EQ-5D-5L index, and reported problems with mobility and self-care) were weaker than expected (hypothesis 8–9, 13–14). This was also the case for the correlation between the symptom index abdominal pain and the EQ VAS score (r = −0.43) (hypothesis 27), which was expected to be strong. In total, 16 (75%) of the 21 hypotheses in the assessment of convergent validity were supported (Tables 1 and 4).

**Table 4:** Spearman’s rank correlations between EQ-5D-5L variables and the other measures

	EQ-5D-5L						
	Mobility (n = 9769)	Self-care (n = 9769)	Usual activities (n = 9769)	Pain/discomfort (n = 9769)	Anxiety/depression (n = 9769)	EQ VAS (n = 8285)	Index value (n = 9769)
PGA score (n = 9651)	0.13	0.09	**0.33**	**0.35**	**0.30**	−0.35	−0.36
SHS 1: Symptoms (n = 9769)	0.25	0.16	0.47	0.57	0.43	**−0.52**	**−0.57**
SHS 2: Interference in daily life (n = 9769)	0.30	0.19	**0.54**	0.55	0.47	**−0.54**	**−0.60**
SHS 3: Worry caused by IBD (n = 9769)	0.21	0.15	0.43	0.47	**0.58**	**−0.50**	**−0.56**
SHS 4: General feeling of well-being (n = 9769)	0.38	0.24	0.54	0.57	**0.57**	**−0.70**	**−0.66**
SI: Abdominal pain (n = 8132)	0.27	0.18	0.43	**0.59**	0.43	−0.43	**−0.57**

All correlations are significant at the 0.01 level (2-tailed). Correlations in grey fields are those that were included in the hypotheses and those in bold were in line with the predetermined hypotheses

*PGA* Physician Global Assessment, *SD* Standard deviation, *SHS* Short Health Scale, *SI* Symptom index

## Discussion

### Discussion of findings

In this study, the known-groups validity and convergent validity of EQ-5D-5L have been investigated in patients with IBD in Sweden and the findings support the construct validity of EQ-5D-5L in this patient group. Sixteen of the 21 hypotheses in the tests of convergent validity and six of the seven hypotheses in the tests of known-groups validity were supported. In total, 79% of the hypotheses were supported, which is above the applied criterion of at least 75% [40]. The study adds to the scarce literature on the validity of the five-level version of EQ-5D in this patient population, and it is the first of its kind using such a large sample with data on HRQoL from a real-world clinical setting among patients with severe IBD in Sweden. It is also the first study of the validity of EQ-5D-5L among patients with UC.

The findings regarding known-groups validity are in line with the few previous studies of EQ-5D-3L and EQ-5D-5L, which have also shown that EQ-5D can detect meaningful differences between patients with active and inactive IBD [23–25]. Further, our study has complemented the previous literature by showing meaningfully important and statistically significant differences in the mean EQ-5D-5L index (UK crosswalk value set) and EQ VAS scores between the four PGA groups (no, mild, moderate, and severe disease activity). In contrast to the results of the previous studies examining EQ-5D-3L, which indicated mainly strong correlations between the EQ VAS and the EQ-5D-3L index value and the disease activity indices (CDAI and CAI), our study showed only moderate correlations between PGA and the EQ-5D-5L index and EQ VAS. Still, the correlations in our study were stronger than the weak correlations between EQ-5D-5L and the physician-reported measures (PDAI and CDAI) reported by Rencz et al. [23]. The differences between the studies may be explained by the use of different activity indices or differences in the composition of the patient populations.

Study findings are consistent with the previous literature indicating that while EQ-5D-5L seems to include some relevant dimensions, mobility and self-care may not be as relevant for patients with IBD [23–25]. The effect sizes between patients with active and inactive disease were lower in the dimensions of mobility and self-care than in the other dimensions. Further, the correlations between the responses in mobility and self-care dimensions and the IBD-specific measures were weaker than between the other EQ-5D-5L dimensions and the IBD-specific measures. As EQ-5D-5L is a generic health outcome measure used to evaluate health status across a wide array of different diseases and conditions, all questions are not necessarily relevant for all patients, but for comparison with other diseases these questions are still important to include as long as some of the other questions are relevant for the patient group.

Interestingly, there were also several correlations that were stronger than expected. The EQ-5D-5L dimensions of Usual activities, Pain/discomfort and Anxiety/depression had at least moderate correlations with all of the other measures, even though not all were included in our hypotheses. It could be argued that some of these correlations should have been anticipated. For example, as pain is an important symptom of IBD, it could be argued that we should have included a strong correlation between the SHS item about symptoms and the dimension of pain/discomfort in the EQ-5D-5L. However, if this correlation (and the other ones) would have been included in our hypotheses, it would only have strengthened the support for the construct validity of EQ-5D-5L, and not changed any of the conclusions.

Previous studies have shown the proportion of patients reporting full health on EQ-5D-3L (i.e., no problems in all dimensions) to be 31% for patients with CD and 43% for patients with UC [25], and 44% for patients with IBD in general [24]. The current study showed a smaller corresponding proportion for EQ-5D-5L for the entire sample (29%). Whilst our results may imply that the EQ-5D-5L may not be sensitive enough to capture difference in health at the healthier end of the scale, it can also be interpreted as an improvement over the EQ-5D-3L. This was also seen in the study by Rencz et al., which showed that the percentage of patients that reported full health decreased from 30% to 26% when comparing EQ-3D-3L to EQ-5D-5L [23].

### Methodological considerations and limitations

An important limitation of our study is that the assessment of construct validity is dependent on the validity of the measures that EQ-5D-5L is being compared to, i.e., the validity of the disease-specific PGA, SHS, and the symptom index abdominal pain. While the validity of the SHS can be supported by previous studies [32, 33], there is less support for the validity of the symptom scales and the PGA used by SWIBREG. There are many different disease activity indices used for assessment of disease activity in patients with IBD and the assessments by physicians have been argued to be subjective [36, 37]. Nevertheless, the PGA registered in SWIBREG stems from the Mayo score, which is one of the most frequently used activity indices in clinical trials among patients with UC [36, 37]. It is also used as a quality indicator in SWIBREG. Further, we consider it a strength of our analyses that we have been able to compare the results from EQ-5D-5L with those of several other measures (i.e., PGA, SHS, and the symptom index about abdominal pain) and believe that the fact that the results from these comparisons go in the same direction strengthen the interpretation of our findings. Data on other disease activity indices, such as the Mayo score, the Walmsley score and the Harvey-Bradshaw Index (HBI) are also collected by SWIBREG. However, when designing the study, all relevant measures were evaluated based on their measurement properties and on the completeness of the data in the registry. As the other indices did not have the same completeness in the registry as the PGA score, it was decided to not include these indices in our analyses.

Another point of consideration is the use of the UK cross walk value set when calculating the index values for EQ-5D-5L in our base case analysis [27]. Recently, a Swedish value set for EQ-5D-5L based on the valuation protocol developed by the EuroQol group was published [28] and there are additional Swedish value sets for EQ-5D-3L and EQ-5D-5L based on experience-based valuations [29, 47]. However, the UK value set for EQ-5D-3L has for long been used in assessments informing reimbursement of pharmaceuticals in Sweden, and the Swedish value set for EQ-5D-5L based on the EQ-VT protocol was not published at the time of formulating the hypotheses. Thus, our hypotheses and the criteria for meaningful important differences (MID) were based on MID values from the UK crosswalk value set. To investigate the influence of the choice of value set, analyses based on the Swedish value sets were added in a sensitivity analysis towards the end of the study. The findings from these analyses suggest that the EQ-5D-5L can detect differences between patients with active and inactive disease even when other value sets are applied. However, to make accurate comparisons of the mean index values from these value sets with a MID estimate, new MID estimates based on these value sets would need to be estimated.

There are multiple biases that could impact the study. Firstly, whilst it is important that the sample size is large enough to be able to reach representativeness and reach statistical significance, large samples can also lead to biased results. This is because large samples are able to produce statistically significant results even when a meaningful effect is not present [22, 48]. To avoid this kind of bias, the study did not focus only on statistical significance, and have reported effect sizes in the analyses of known-groups validity and the strength of correlations in the analysis of convergent validity. However, whilst there have been various attempts to estimate a MID for the EQ-5D, there is no agreed upon value. Based on the results of a previous study among patients with IBD, we have in this paper used a value of 0.08 for the EQ-5D index value and 11.0 for EQ VAS [25]. These MID values were estimated from changes in the EQ-5D index value among patients with IBD that had reported that their health status had improved or worsened, A very similar general MID estimate (0.08) for the UK EQ-5D-3L index has been presented in a study based on an instrument-based approach [49]. Another study among patients with active Crohn’s disease has found a lower MID (9.2) for EQ-VAS, which suggests that even smaller differences in EQ-VAS could be considered meaningful [50].

Secondly, multiple comparisons bias can arise when testing multiple hypotheses, as the probability of finding at least one significant result increases with the number of hypotheses being tested. To prevent this type of bias it has been recommended that at least 75% of the hypotheses should be satisfied in order to conclude that a measure is valid [40]. In this study, 79% of the pre-defined hypotheses were supported.

Another issue that needs to be considered is that we have used measurements from a time period of nine years. Thus, differences in diagnostics, treatment or other patient characteristics may have changed over time and may have had an impact on the results. To investigate this, differences over time were analysed by presenting the data in box plots and histograms for each year. This analysis showed no meaningful differences in any of the variables used in the study over time indicating that there had been no apparent systematic changes in the HRQoL of the population over time.

### Implications

The results of this study have implications for researchers, healthcare professionals, patients and other decision makers who collect or use results from HRQoL measures from patients with IBD. Ensuring that there is support for the validity of the HRQoL measures that are used in routine care or in clinical studies is essential to be able to draw valid conclusions from the results [19, 51]. Thus, sufficiently robust measurement properties are included as a requirement in several guidelines for how to select the most appropriate PROM and for the implementation of PROMs in clinical practice [51–54]. In this context, studies of the measure’s construct validity have an important role to play along with studies of reliability and responsiveness. As the validity of an instrument may vary between different populations, the validity needs to be confirmed in the specific patient population [19, 20]. For quality registries such as SWIBREG, which collect PROM data from a large number of patients in routine health care, the validity of the included measures is important to ensure that the results accurately describe the patients’ perception of their health and can detect important differences in health or HRQoL. HRQoL data from valid generic measures can be used to assess patient population needs and health care from a more holistic perspective than the disease-specific instruments [55]. Furthermore, the results can be compared with those from other disease areas. The use of generic measures can thus complement the disease-specific measures and can have a positive impact on the future care of this patient group. In contrast, the use of a measure with poor validity among patients with IBD, which cannot detect important differences in HRQoL, may lead to biased results that falsely suggest that treatments or other interventions aimed at patients with IBD have no effect or may lead to underestimates of the impact of the disease on the patients’ HRQoL compared to other patient groups [19]. Thus, the results of this study, which support the validity of the EQ-5D-5L among patients with IBD, may have important implications for the selection of HRQoL measures when collecting data in routine health care. Likewise, the findings have implications for future clinical or health economic studies considering using the EQ-5D-5L as a measure of HRQoL in this patient population.

## Conclusion

In conclusion, the analyses in this paper support the construct validity of the EQ-5D-5L amongst patients with IBD. These results are in line with previous studies but are based on a significantly larger sample, adding to the scarce literature on the validity of the five-level version of EQ-5D in this patient population. The findings supporting the use of EQ-5D-5L amongst patients with IBD have important implications for the collection of PROMs in routine health care registries like SWIBREG. Further, the results have implications for the choice of HRQoL measure in future clinical or health economic studies considering using EQ-5D-5L as a measure of HRQoL in this patient group.

## Data Availability

Access to data is restricted by Swedish law. General information about obtaining access to data is available from the corresponding author Emelie Heintz upon request.

## List of abbreviations

CD: Crohn’s disease
EQ VAS: EQ visual analogue scale
HRQoL: Health-related quality of life
IBD: Inflammatory bowel disease
IBD-U: Unclassified IBD
MID: Minimally important difference
PGA: Physician global assessment
PROM: Patient-reported outcome measure
SD: Standard deviation
SHS: Short health scale
SWIBREG: Swedish Inflammatory Bowel Disease Registry
UC: Ulcerative colitis
